# Assessing the impact of household participation on satisfaction and safe design in humanitarian shelter projects

**DOI:** 10.1111/disa.12405

**Published:** 2019-08-21

**Authors:** Aaron Opdyke, Amy Javernick‐Will, Matt Koschmann

**Affiliations:** ^1^ Lecturer, School of Civil Engineering University of Sydney Australia; ^2^ Associate Professor, Department of Civil, Environmental, and Architectural Engineering University of Colorado Boulder United States; ^3^ Associate Professor, Department of Communication University of Colorado Boulder United States

**Keywords:** housing, participation, Philippines, qualitative comparative analysis, shelter, Typhoon Haiyan

## Abstract

Participation has long been considered important for post‐disaster recovery. Establishing what constitutes participation in post‐disaster shelter projects, however, has remained elusive, and the links between different types of participation and shelter programme outcomes are not well understood. Furthermore, recent case studies suggest that misguided participation strategies may be to blame for failures. This study analysed 19 shelter projects implemented in the Philippines following Typhoon Haiyan in November 2013 to identify the forms of participation employed. Using fuzzy‐set qualitative comparative analysis, it assessed how household participation in the planning, design, and construction phases of shelter reconstruction led to outcomes of household satisfaction and safe shelter design. Participation was operationalised via eight central project tasks, revealing that the involvement of households in the early planning stages of projects and in construction activities were important for satisfaction and design outcomes, whereas engagement during the design phase of projects had little impact on the selected outcomes.

## Introduction

The United Nations estimated a funding gap of USD 15 billion for humanitarian needs in 2015 (Georgieva et al., 2016). This deficit is particularly prevalent in the shelter and settlements sector, which historically has relied on delivering outputs that are costly.^1^ As Graham Saunders pointed out: ‘The scale of post‐disaster shelter need that is increasingly emerging is beyond the response capacity of institutional humanitarians, be they governmental or non‐governmental’ (Davis, 2011, p. 203). The result is a growing emphasis on supporting ‘self‐recovery’ and homeowner‐driven models of shelter and housing reconstruction (Maynard, Parker, and Twigg, 2017). These approaches will become the new norm for responding to disasters. The debate surrounding the benefits, pitfalls, and realities of participation in humanitarian shelter programming is thus becoming increasingly important as humanitarian funding is stretched to meet a mounting number of conflicts and disasters triggered by natural hazards.

Participation, which emerged from neoliberal policies and the democratisation of aid, has become a pillar of disaster assistance (Pyles, 2011). At its core, the participation of affected households and local governments has been associated with empowerment (Chambers, 1997), cost reduction (Ferguson and Navarette, 2003), decentralisation of governance (Ahrens and Rudolph, 2006), and local knowledge (Hayles, 2010). Yet, despite its central reoccurring role in disaster discourse, policy, and theory, participation in disaster recovery remains an ambiguous narrative, the product of vague operational definitions and the misrepresentation of consulting and informing as legitimate forms of participation (Davidson et al., 2007).

Entangled in efforts to support recovery, participation has taken on a plethora of definitions that are frequently derived from theoretical notions, rather than practical observations from disaster contexts. Furthermore, the causal links between participation and shelter outcomes, both positive and negative, are too regularly anecdotal, and while temporality has become of emerging significance in disaster scholarship (see, for example, Olshansky, Hopkins, and Johnson, 2012), past research on participation often neglects the important question of *when* different types of participation occur (that is, during what project phase). Clarifying and operationalising participation in humanitarian shelter and settlement projects, as well as understanding causal links to project outcomes, can better inform how governments and non‐governmental organisations (NGOs) approach shelter assistance.

This study echoes calls made nearly 40 years ago by Cohen and Uphoff (1980) for ‘clarity through specificity’ of participation. In place of generalities, it is imperative that one comprehends participation as specific tasks that are situated within a project cycle. To date, much of the literature on participation in shelter poorly defines what actually constitutes participation, and by whom, resulting in a spectrum of definitions and practices that are loosely associated. Hence, this paper unpacks types of participation observed in post‐disaster shelter projects to address the following research question (RQ1):

- *What types of household participation occur in post‐disaster shelter projects?*

Operationalising participation in post‐disaster shelter projects is necessary to understand whether participation leads to positive or negative shelter outcomes, including when it occurs (Prokopy, 2005). While the lack of causality between participation and shelter outcomes is partially associated with a lack of consensus on what constitutes and defines participation in shelter projects, it also stems from limited cross‐case analysis within the field. A second question was formulated to address this fact (RQ2):

- *How, and when, do different types of participation affect post‐disaster shelter outcomes?*

To explore participation, the study examined shelter projects following Typhoon Haiyan in the Philippines in November 2013, which damaged or destroyed nearly 1.1 million houses, and led to a large international humanitarian response. Haiyan is a compelling case to evaluate because of the large variation in approaches that emerged among implementing organisations in the shelter and settlement sector. This paper first provides background on literature on shelter outcomes, participation, and the tenuous connection between participation and shelter outcomes in post‐disaster shelter programmes. Next it discusses the methods employed to identify types of participation, as well as the procedure adopted to analyse the causal links between participation and shelter outcomes: fuzzy‐set qualitative comparative analysis (fsQCA). Lastly, it sets out and discusses the results.

## Background

The study first reviewed shelter outcomes, focusing on household satisfaction with shelter and technically sound shelter designs. Subsequently, it assessed participation within post‐disaster shelter programmes and existing work that has connected participation and shelter outcomes.

### Shelter outcomes

Shelter is universally recognised as a foundational aspect of disaster recovery. While its ability to provide protection from the elements is a core function, shelter also contributes to re‐establishing household routines (Quarantelli, 1982; Peacock, Dash, and Zhang, 2007), simulating economic activity (Sheppard and Hill, 2005) and restoring social ties (Mileti, 1999). Previous literature has linked shelter to these specific benefits, as well as to broader recovery (see, for example, Jordan and Javernick‐Will, 2013) and resilience outcomes (see, for example, Kusumastuti et al., 2014; Cutter, 2016). In practice, organisations have too often relied on coverage, such as the number of households assisted, to gauge the impact of shelter assistance, neglecting to evaluate whether or not shelter assistance actually fulfils its intended purpose. However, numerous indicators have emerged to measure the *quality* of shelter project outcomes (see, for example, Nath et al., 2017).

Drawing on the work of Jha et al. (2010), this study opted to scrutinise two outcomes that demonstrate the functionality of shelter in meeting household needs and reducing risk in the future: household satisfaction; and safe shelter design. The satisfaction of beneficiaries remains the most common determinant of the success of shelter projects (Piccioli et al., 2017). Safe design, by contrast, is an understudied but vital component of resilience (Bruneau et al., 2003).

#### Household satisfaction with shelter

Satisfaction with shelter has been applied consistently as a means of assessing the ability of shelter to satisfy household needs. For instance, Snarr and Brown (1980) noted its ability to appraise how well housing serves its function, departing from earlier measures that concentrated on the number of shelters completed. Barenstein (2009) used a similar measure to compare the Latur and Gujarat earthquakes in India on 30 September 1993 and 26 January 2001, respectively, and the Indian Ocean tsunami on 26 December 2004 (in the Indian context), and Rand, Hirano, and Kelman (2011) used the outcome ‘satisfaction’ after the Indian Ocean tsunami of 2004 as a means with which to judge the ability of shelter to meet household needs. In Bouraoui and Lizarralde's (2013) compilation of shelter satisfaction indicators, one‐third of the pointers identified focused on comparing existing housing, infrastructure, and services to the pre‐disaster state. In assessing the outcomes of shelter *projects*, comparison to a pre‐disaster state provides analogous data that can be considered across disaster contexts. As such, this study followed similar investigations, such as that of Barenstein (2006), and opted to measure household satisfaction according to average perceptions of current shelter as compared to original dwellings.

#### Shelter designs

Poorly constructed shelter is regularly the cause of death in disasters. In addition, access to safe shelter is identified as a key outcome in the *Sendai Framework for Disaster Risk Reduction 2015–2030* (UNISDR, 2015). Building codes offer an ideal standard for design but are often beyond the reach of households in resource‐limited communities. Thus, determining what constitutes safe shelter design is difficult and frequently highly dependent on local construction methods and materials. Previous research has assessed the safety of post‐disaster shelter largely through a comparison with previous conditions, including Arlikatti and Andrew's (2012) evaluation of shelter construction in India after the tsunami of 2004.

In recent years, the Global Shelter Cluster, an Inter‐Agency Standing Committee coordination mechanism, has created key messages to promote safer shelter design and construction in the wake of disasters. Following Typhoon Haiyan in the Philippines, it produced ‘eight build back safer key messages’ that proposed design recommendations for households in relation to the following themes: bracing; foundations; joints; roofing; preparation; shape; site location; and tie‐downs. This study looked at seven of the eight key messages to gauge the presence, or absence, of safe shelter design, omitting ‘preparation’ as it is not related to shelter design. In contrast to past studies, which have spotlighted building materials or visual signs of deficient construction quality, the approach used here systemically analysed structural details to ascertain adherence to the eight key messages. Full details of the assessment methods can be found in Opdyke (2017).

### Participation within post‐disaster shelter programmes

Participation has become so institutionalised in practice that it is unequivocally accepted as necessary in shelter projects. The abundance of titles pertaining to participation symbolises how dispersed theory has become over decades of research, notably ‘citizen participation’, ‘community participation’, ‘popular participation’, and ‘user participation’ (Arnstein, 1969; Cornwall, 2006; Davidson et al., 2007; Sadiqi, Trigunarsyah, and Coffey, 2017). Most conceptualisations of participation stem from broader planning or development literature, such as Arnstein's (1969) ‘ladder of citizen participation’, the framework for which was later adapted by Choguill (1996) for underdeveloped countries. By adopting these theoretical frameworks of participation, however, there has not been a full examination of how participation surfaces in disaster practice, and, accordingly, specificity has been lost. Many previous studies have neglected, therefore, to define the type of participation that they analyse; among those that do, there remains little consensus on a definition.

The application of planning and development definitions of participation have fixated on decision‐making as the focal point, discounting other forms of participation, such as sweat equity, as token forms. Vallance (2015) adeptly points out that participation in implementation, such as sweat equity, is often falsely used as a proxy for participation; little research, though, has tried to examine multiple types of participation in parallel. Participation should be understood for what it is: a graded scale of decisions *and* actions (Lawther, 2009).

As Davidson et al. (2007, p. 102) note, ‘community participation in disasters has not been defined in terms of what it means in a project environment’. Thus, there remains a lack of an organised framework of project tasks, both decision‐ and implementation‐based, that can be used to measure participation in shelter projects. This study proposes that participation can be defined as household inputs into shelter projects. The operationalisation of participation is approached via a grounded perspective that assesses project tasks in shelter planning, design, and construction.

### Links between participation and shelter outcomes

There are myriad shelter case studies suggesting that participation is an essential component of successful shelter projects (Barakat, 2003). The former Office of the United Nations Disaster Relief Coordinator (UNDRO, 1982, p. 55) went as far as to state that: ‘The key to success ultimately lies in the participation of the local community – the survivors – in reconstruction’. However, a closer examination of the literature reveals that understanding of the linkages between participation and shelter outcomes is less than conclusive. In a review of broader community‐based development research, Mansuri and Rao (2004) found no studies that identified a causal connection between outcomes and participatory project elements. Evidence from past post‐disaster shelter research indicates that community involvement is necessary; however, full community control may not be needed to achieve outcomes, such as satisfaction (Kennedy et al., 2008).

Both Bouraoui and Lizarralde (2013) and Rand, Hirano, and Kelman (2011) pinpointed a positive association between participation in shelter projects and satisfaction of end users, with the latter reporting that participation during the *construction* phase was linked to user satisfaction. Yet, there is relatively little evidence concerning the impact of participation on safe shelter design. One study by Khwaja (2004) discovered a negative relationship between community participation in decision‐making and infrastructure design outcomes, although this was not specific to shelter projects or the disaster setting.

From these examples, one can see that the effect of participation on shelter outcomes varies greatly, with the relation between them contingent largely on the shelter outcome and the type of participation analysed. For instance, satisfaction has previously been evaluated in relation to decision‐making participation during construction, but no studies were unearthed that explicitly addressed other types of participation, such as labour, with respect to this outcome. There are also methodical gaps in the literature that have hindered understanding of the links between participation and shelter outcomes. Specifically, despite a strong foundation that supports a dynamic, non‐linear understanding of recovery processes in the realm of disaster literature (see, for example, Smith and Wenger, 2006), the importance of when participation occurs during recovery has largely been neglected, and few studies have investigated shelter recovery in a longitudinal manner (Snarr and Brown, 1980, 1982, 1994). Hence, there is a need to contextualise the use of participation within longitudinal studies to understand how involvement during planning, design, and construction influences shelter outcomes (Peacock, Dash, and Zhang, 2007; Kelman et al., 2011). To meet this need, the study focuses on identifying types of participation across post‐disaster shelter project phases, and assessing the impact of this participation on the shelter outcomes of household satisfaction and shelter design.

## Methods

To address the two research questions, the decision was taken to explore post‐disaster shelter projects, defined here as shelter assistance provided by a single organisation within a barangay, the lowest political division in the Philippines—the research context. While thousands of households might receive shelter assistance, a small number of programmes are responsible for assisting these masses. For instance, in response to Typhoon Haiyan in the Philippines, the Global Shelter Cluster (2014a) reported that 71 organisations were responsible for assisting 344,853 households. Although this number is sufficient for considering statistical means of comparing programmes, the collection of data of sufficient detail is prohibitive. As a result, case studies have become the norm for the investigation of post‐disaster shelter projects. The value of case study research in disasters should not be discounted, but its core limitation is its ability to generalise. Recognising the limits of past work on participation, this study sought to examine a larger number of cases within a single disaster setting, the Philippines, using fsQCA. A notable characteristic of this analytical technique is ‘equifinality’, or the concept that an outcome can be achieved in different ways, allowing different combinations of conditions to lead to the result of interest. Thus, fsQCA is a cross‐case analytic procedure that permits the construction of causal models, facilitating an understanding of what conditions, in what combination, lead to a desired outcome (Ragin, 1987).

The paper first describes the research context and the data collected, before discussing the analysis and the results with respect to the two research questions. During the first phase, the study aimed to create a typology of participation and develop a set of conditions that could be employed to assess their impact on the selected outcomes. During the second phase, the study examined the links between participation and the shelter outcomes of household satisfaction and safe shelter design.

### The research context

The study focused on post‐disaster participation and outcomes following Typhoon Haiyan in the Philippines in November 2013, which affected more than 16 million people and was responsible for damaging or destroying more than 1.1 million homes in its path (Global Shelter Cluster, 2014a). In consultation with shelter organisations involved in the response and recovery, 19 shelter projects were chosen for analysis over a three‐year period. The study selected projects in communities that had experienced extensive damage, and that received shelter assistance from different implementing organisations, which had varied approaches to participation. Of the communities chosen, one‐third of them were in urban environments, whereas the remainder were either peri‐urban or rural, broadly reflecting the location of populations affected by Haiyan. A range of urban and rural communities was intended to compare universal concepts of participation processes. Furthermore, all of the projects were selected during the planning stages prior to the start of substantial design or construction activities, in order to follow each project through all cycles. More than one province was chosen in case province was an important factor. The provinces of Cebu, Leyte, and Eastern Samar were picked because they suffered the most damage, and thus had the greatest need of shelter, and because they were close enough to allow for the completion of the intensive data collection exercise required.

Table 1 contains a list of the communities selected and shelter assistance details. Each project is categorised by the type of shelter assistance provided. Repair and retrofit programmes upgraded structures with minor damage; transitional shelter served as an interim solution for relocated households; and core/progressive shelter provided a basic structure that could be expanded over time. Rental subsidies supplied cash to renters; hosting support gave joint family living arrangements access to cash; and resettlement projects involved construction at new sites, often distanced from previous coastal hazards. Households receiving shelter assistance from other organisations outside of the primary project considered within a community were excluded. In one community, for instance, three organisations were offering households shelter assistance; the analysis was bound only to those households receiving assistance from the organisations identified for inclusion in the study. For each of the shelter projects selected, interview, documentation, and observation data were collected during field visits at periods of 6, 12, 28, and 36 months after the disaster.

**Table 1:** Project and community overview

Case	Community	Municipality	Province	Population	Households assisted	Shelter categories∗
1	Okoy	Santa Fe	Cebu	3,532	230	3
2	Maricaban	Santa Fe	Cebu	2,999	118	6
3	Poblacion	Santa Fe	Cebu	2,345	40	3, 6
4	Sungko	Bantayan	Cebu	3,296	183	1, 2
5	Sillon	Bantayan	Cebu	4,064	75	3
6	Kangkaibe	Bantayan	Cebu	2,635	348	3, 6
7	Tagpuro	Tacloban City	Leyte	677	86	2
8	Pago	Tanauan	Leyte	917	365	6
9	New Kawayan (101)	Tacloban City	Leyte	543	148	1
10	Bagacay (93)	Tacloban City	Leyte	3,936	150	3
11	San Agustin	Jaro	Leyte	824	45	3
12	San Jose (83C)	Tacloban City	Leyte	2,548	42	3
13	Magallanes (52)	Tacloban City	Leyte	1,304	199	1, 2, 3, 4, 5
14	San Jose (85)	Tacloban City	Leyte	1,572	234	1
15	Hiabangan	Dagami	Leyte	958	165	1, 3
16	Sagkahan (62)	Tacloban	Leyte	1,434	484	1, 3, 4, 5
17	Sulangan	Guiuan	Eastern Samar	3,597	63	1, 3
18	Cogon	Guiuan	Eastern Samar	1,146	133	2, 6
19	Cantahay	Guiuan	Eastern Samar	1,118	105	3

**Notes:** ∗ The shelter categories are as follows: 1. Repair and retrofit; 2. Transitional shelter; 3. Core/progressive shelter; 4. Rental subsidies; 5. Hosting support; and 6. Resettlement. **Source:** authors.

### Data collection

During the first field visit, spanning four months, 32 semi‐structured interviews were held with NGO staff, local government officials, and community members involved in selected communities. Participants stemmed from international and domestic NGOs, local government units (LGUs), the Global Shelter Cluster, and the Global WASH (Water, Sanitation, Hygiene) Cluster.

Interview questions during this initial round of fieldwork centred on understanding how organisations involved, or did not involve, households in the early planning and design of shelter assistance. Examples of interview questions presented to organisations and households, respectively, are: ‘how are you involving beneficiaries in your shelter projects?’; and ‘how are shelter designs being determined?’. In addition to interviews, field notes were recorded based on daily observations of reconstruction projects, cluster coordination meetings, and internal organisation meetings. These notes encompassed dialogue that occurred during meetings and observation of stakeholder interactions in on‐site planning activities. Finally, cluster policy documents, meeting minutes, recovery plans, and technical communication documents were gathered.

A second three‐month field visit was conducted four months later, during which an additional 167 interviews were performed with stakeholders. Individuals were selected based on continuing reconstruction efforts as part of projects identified during the first phase. Questions again focused on the types of participation occurring; however, participation in the design and construction phases was emphasised. Example questions included: ‘what is being requested of beneficiaries during construction?’; and ‘what were you asked to contribute?’.

The third three‐month field visit took place after the completion of the selected shelter projects. In‐person surveys were used to procure data on shelter project outcomes. In total, 320 surveys across the 19 shelter projects were administered. Relevant questions included asking households to evaluate their current shelter as compared to their dwelling before the typhoon, and to make a visual assessment of the structural characteristics of shelters. These questions were presented verbally using a translator, similar to the semi‐structured interviews, and responses were recorded using a tablet computer.

A final two‐week field visit served to follow up on missing data and to triangulate conflicting information through 12 additional interviews with organisation staff and households.

### Phase 1: operationalising participation in post‐disaster shelter

#### Data analysis

All of the interviews were translated and transcribed before being imported into NVivo (QSR International) qualitative coding software where the data were inductively coded into participation themes. Coding was completed independently by two researchers prior to inter‐coder comparison testing to verify themes in the data (Campbell et al., 2013). After themes were determined, inter‐rater reliability scores in the form of Cohen's Kappa coefficient were computed for comparison among a 20 per cent sample of interviews. Kappa coefficients, statistical measures of intercoder reliability, are a more robust measure than simple agreement measures because they consider the amount of agreement between coders that is likely to occur by chance. Values in excess of 0.4 are generally seen as acceptable (Landis and Koch, 1977). If this threshold was not met for the coding of any interview, the two researchers revisited the coding to reach consensus. Coding queries were then used to summarise themes across projects for each condition.

#### Results

The qualitative analysis revealed eight conditions that characterised participation in shelter projects, which were subsequently categorised into the planning, design, and construction phases of projects: (i) determination of aid and (ii) location selection (planning conditions); (iii) floorplan and layout and (iv) government permitting (design conditions); and (v) sweat equity, (vi) material procurement, (vii) financial management, and (viii) oversight (construction conditions). These are defined in Table 2 and described further below.

**Table 2:** Condition definitions

	Condition	Definition
Planning	Determination of aid	The involvement of households in formal needs assessment processes, either through a third party or the implementing shelter organisation.
	Location selection	The ability of households to have agency in deciding the site of their shelter.
Design	Floorplan and layout	Household have the ability to control decisions on the layout and design of their shelter.
	Government permitting	Formal documented approval by the local municipality or city regarding the location and design of shelter interventions.
Construction	Sweat equity	Unpaid labour contributions during construction that may consist of either skilled or unskilled tasks.
	Material procurement	Obtaining materials required to complete the construction of the planned shelter.
	Financial management	Household management of financial resources required to complete the shelter, including labour, materials, transportation, and other essential tasks.
	Oversight	The supervision of construction tasks by beneficiary households.

**Source:** authors.

### Planning phase

The first decision observed in shelter projects was who to assist, and where. *Determination of aid* differs by organisation, but was distinguished by whether or not a formal assessment was conducted. Some shelter programmes established needs via third‐party assessments, such as by a government municipality. Combined with reported damage levels, organisations often predetermined shelter approaches, such as repair kits for regions recognised as having suffered minimal damage, limiting the participation of households. Other organisations opted to perform their own assessment, amassing local perspectives before making decisions on how best to implement shelter assistance. Finally, others negotiated with donors to allow communities to determine their own needs before identifying shelter as the best means of assistance. These methods of determining aid were not mutually exclusive and organisations that conducted first‐hand assessments or facilitated community inputs frequently built upon earlier second‐hand reports.

The decision concerning *location selection* was the second task pinpointed during the planning phase, which was pertinent to participation. The coastal ‘no‐build’ zones, defined by the Government of the Philippines as a 40‐metre buffer along coastlines, within which structures could not be built, shaped many location decisions. These relocation sites saw variation in the distances families were moved, yet most were at least a few kilometres away and predominantly green‐field sites that lacked pre‐existing infrastructure and services. As one shelter beneficiary described: ‘Yes, they informed us about the shelter assistance, and that relocation for all those from the no‐build zone is compulsory, for they considered the 40‐metre from the shore as a danger zone’. In some cases, however, organisations sought to provide choice for relocation: ‘During one of the meetings [with the project manager], he left us to decide where we wanted our house in the relocation site. He had with him an illustration of the relocation site and he let everyone identify [in] which region we wanted our house built, the colour of it, and whom we wanted as our neighbours’. While choice was eventually afforded in the later stages of site planning, it is clear that attempts gravitated towards informing rather than placing the decision in the hands of households.

### Design phase

The *floorplan and layout* of shelters were dictated in some cases, whereas other programmes allowed for flexible options so that households could choose the configuration of rooms, windows, and doors. Design also encompassed the critical matter of what materials to use in shelters, as engineers have long advocated for more resilient resources to address risk (Bosher, 2014). Material selection dictated sourcing, cost, and labour, each affecting shelter outcomes uniquely. While material selection might be viewed as separate from the floorplan and shelter layout, these were inseparably linked across all of the cases in this study. There were noticeable differences in participation that either leaned towards consultative processes or control forfeited to households.

The role of local government in shelter projects emerged as an important and complementary type of participation for inclusion. In particular, there were evident differences in project outcomes between high and low levels of government participation that led to the inclusion of this condition within the design phase of projects. *Government permitting* of shelter designs not only allowed for vernacular building features to be incorporated and accounted for in designs, but also yielded institutional protections for shelter assistance, such as recognition of land agreements and land tenure.

### Construction phase

One of the most controversial types of participation, *sweat equity*, has largely been examined in isolation from other types of participation, despite the fact that it is often highly embedded in social norms or modalities of delivering assistance. Common unskilled labour tasks included clearing sites, moving materials, and excavation. In some cases, if a household member had previous construction knowledge, they were asked to participate in technical jobs such as framing walls, masonry placement, and roofing. Requirements for sweat equity ranged from participation that was simply encouraged up to requirements of 2,000 logged hours per beneficiary household.

*Material procurement*, or the acquisition of construction materials, was another construction task that varied across projects. Beneficiaries were observed as either being required to procure materials through designated suppliers or to identify their own suppliers. In other projects, the organisation handled the procurement of materials. In cases where the organisation acquired materials, the most common reason was related to concerns about local quality. In some instances, organisations paid suppliers directly but households were responsible for selecting and transporting materials, thus procurement was not entirely based on a cash transfer to households and is separate from the financial management condition.

*Financial management* by beneficiaries was yet another category of participation that was drawn from the literature and confirmed by field observations. Past research has suggested that owner‐managed reconstruction is cheaper and quicker (Schilderman and Lyons, 2011), making it a valuable condition to include in the subsequent analysis. The most common example of financial management that the study witnessed was associated with conditional cash transfers, where the household was responsible for hiring labour and obtaining needed resources for construction. This necessitated the household overseeing project finances and allocating resources as required to ensure construction activities were accomplished.

Previous research has also noted the increasingly important role of *oversight* during construction. Studies have shown that organisation and household supervision of construction activities ensures quality control of housing and leads to more durable structures (Davidson et al., 2007; Jordan, Javernick‐Will, and Tierney, 2016). Examples of oversight included inspections by both households and the implementing organisations, as well as checklists to verify construction was in compliance with designs.

### Phase 2: causal links between participation and project outcomes

#### Data analysis

In the absence of rigorous small‐N case comparisons in humanitarian shelter research, fsQCA was used to evaluate how, and when, participation of households is important in shelter projects. This technique offers a middle ground between case studies and statistical analysis, retaining complexity within cases, while still offering the ability to generalise findings through robust comparisons (Ragin, 1987). A particular outcome of interest is identified (such as household satisfaction) along with conditions (such as location selection) posited to affect that outcome. The method draws on Boolean algebra and set logic to assess how conditions, in combination or isolation, compose ‘pathways’ to the desired outcome.

#### Variable calibration

Building on the first phase of the research, the study explored eight types of participation and two shelter outcomes that surfaced from shelter projects. A ninth condition, value of aid, was added to account for projects that had substantially higher resources allocated per household, to explain potential differences.

Qualitative comparative analysis relies on a set theoretical approach, which contrasts traditional statistical methods that use correlational measures. The first step was to calibrate the raw data, resulting in preliminary anchor points, membership and non‐membership, for each condition being established. For conditions that were indirectly coded, meaning that the calibration of the condition relied on qualitative sets, a level of precision for each condition was determined based on classifications that emerged from the qualitative coding summaries (Basurto and Speer, 2012). For each condition, qualitative classifications were assigned specific values. Cases with only two classifications were turned into binary, or crisp, sets, where a value of ‘1’ indicated membership within a set and ∗∗∗‘0’ indicated non‐membership. For instance, the calibration for location selection was a crisp set, where a score of ∗∗∗‘0’ was assigned to cases where the households had no say in the location of their shelter, and a score of ‘1’ designating that households were involved in the decision‐making process regarding the location of their shelter.

For participation conditions with a greater number of distinct classifications of cases, a higher number of set scores was used. Table 3 shows an example fuzzy‐set calibration for floorplan and layout during the design phase. A score of ‘0’ was given when households were never consulted. A score of ‘0.33’ was assigned when households were consulted through a large community meeting—this was included as the intermediate out‐of‐set value because meetings would suppress the voice of minorities in communities. As compared to out‐of‐set membership, in‐set membership was distinguished by bidirectional communication between households and the implementing organisation. For in‐set membership, the study further distinguished between receiving input on plans already developed, where homeowners may have withheld views owing to concern about losing aid support (a score of ‘0.67’), and where there was open‐ended dialogue with homeowners to determine features and floorplan designs (score of ‘1’).

**Table 3:** Example of fuzzy‐set variable calibration

Floorplan and layout	
0	Households were never consulted on the floorplan and the layout of the shelter.
0.33	Households were consulted at a large community meeting to discuss housing features.
0.67	Households were provided with a floorplan and asked about their housing design preferences, such as the location of doors and windows, which were then included in the final design.
1	Households were asked to participate actively in the development of floorplans and had control over final design decisions.

**Source:** authors.

The other seven participation conditions were coded following similar steps and using the indirect calibration method. The full list of calibrations can be found in Annexes 1 and 2.

In contrast to the indirect calibration method, the outcomes of household satisfaction and shelter design, as well as the value of aid condition, were calibrated using the direct calibration method. Direct calibrations use interval‐scale data and rely on three qualitative breakpoints to structure the set. The researcher defines full membership (‘0.95’), the crossover point (‘0.5’), and full non‐membership (‘0.05’). These theoretically defined points are then used to transform the original interval‐scale data into a fuzzy scale using transformations that utilise the log odds of full membership (Ragin, 2009).

Drawing on the example of value of aid, the first step was to set breakpoints using theoretical and case knowledge. As part of data collection, the study determined the average monetary value of assistance provided to households for each shelter project. Anchor points were then defined using estimates compiled by the Global Shelter Cluster (2014b): PHP 20,000 for out‐of‐set membership, aligning with the expected cost of major repairs; and PHP 185,000 for in‐set membership, aligning with the expected cost of a permanent shelter. The point of maximum ambiguity was set at PHP 85,000 as this estimate was for a ‘core’ shelter that did not include basic components, such as a kitchen or a latrine, and was thus designated as the crossover point. Using these anchor points, log odds were employed to calibrate the sets and to assign fuzzy values to each case.

#### Analysing causal conditions

After calibrating the selected conditions and outcomes, a truth table was compiled and the analysis conducted using fsQCA software (Ragin, Strand, and Rubinson, 2008). The full truth table used for the analysis is shown in Annexe 3. Qualitative comparative analysis relies on two primary measures to assess ‘causal recipes’ of conditions: consistency; and necessity. Consistency is the degree to which one condition (or combination of conditions) is a subset of another condition (Jordan et al., 2011). The second measure, necessity, considers whether an outcome is composed of a subset of instances of a particular condition. The term ‘coverage’ is often substituted by necessity when discussing combinations of conditions in a solution. The equations used to calculate consistency and necessity are shown below:



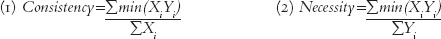



Acceptable values of consistency are typically ‘0.8’ for sufficient conditions (or combinations of conditions), whereas necessary conditions are those with a value of ‘0.9’ or more (Ragin, 2008).

In qualitative comparative analysis, the logic space is defined as all of the possible value combinations of conditions (Ragin, 1987). To reduce the study's logic space simplifying assumptions were made regarding the expected theoretical direction of relationships between each condition and outcome (Ragin and Sonnett, 2005). A list of simplifying assumptions is provided in Annexe 4. For instance, one would expect allowing households to select the location of their shelter to lead to greater household satisfaction. Following a preliminary screening of individual condition necessity with respect to the three outcomes, a subset/superset analysis was performed, in a bid to reveal if there were any conditions that can be removed from solutions. This step examines the consistency of groups of conditions to identify common denominators. The product was final causal pathways that describe different combinations of participation resulting in the presence, or absence, of household satisfaction with shelter design.

## Findings

The findings for each outcome are discussed individually and then collectively in relation to themes identified across the outcomes and projects. The following subsections contain the solutions, or combinations of conditions, identified for each outcome. A ‘∼’ denotes the absence of a condition, and a ‘∗’ denotes the ‘and’ Boolean operator.

### Shelter satisfaction

**Figure 1:**
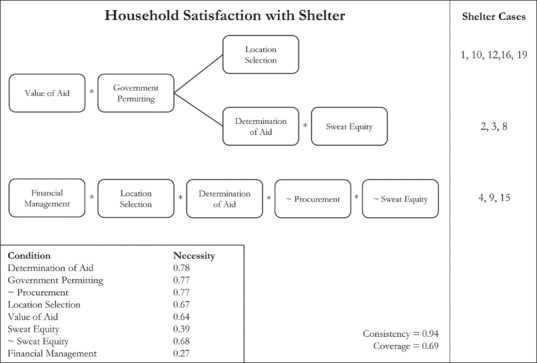
Household satisfaction pathways **Source:** authors.

Shelter projects broadly met with high levels of satisfaction, measured using a Likert scale ranging from ‘much worse’ to ‘much better’ in comparison to pre‐disaster shelter. Variation was still noticed in the levels of satisfaction achieved however, which were investigated using identified participation conditions. Thirteen of the shelter projects showed signs of household shelter satisfaction and were included in the outcome membership set, whereas six projects exhibited low satisfaction. From the analysis, three participation pathways surfaced with an overall consistency of ‘0.94’ and a coverage of ‘0.69’. Figure 1 below shows the pathways to household satisfaction with shelter.

For the first two pathways found, core components included a high value of aid and government permitting of shelter plans. The importance of access to a sufficient value of aid was described by one household when asked if the materials being used in reconstruction were better than those used prior to the typhoon:

*It depends, because those who are in the higher income brackets can afford to buy good‐quality materials, while those who earn less just settled for the ordinary materials. If we opt to use good lumber, the allocated budget for the materials will be insufficient, so we had to settle with what can suffice with the resources available*.

In addition, either location selection or a combination of determination of aid and sweat equity was also required. The first of these pathways covered five of the 13 cases that showed high satisfaction; the second pathway covered three cases. Households frequently noted that their satisfaction with shelter was often a product of where they were allowed to build. For instance, one relocated beneficiary who was dissatisfied stated that: ‘We do not have transport service to go fishing again’. This causal link between household satisfaction and location is well established in the literature (Rumbach, 2014), yet programmes continue to neglect both the economic and social dimensions of shelter location.

Interestingly, it is clear that all three of the cases included in the second pathway were relocation projects, where participants were not involved in selecting the location of their house. This suggests that participation in early needs assessments and sweat equity were able to substitute for location decision‐making in generating satisfaction. While resettlement should be considered only as a last option, it may be necessary in select cases. The third pathway covered three cases, but is distinguished by greater control over financial resources. The pathway also included location selection, determination of aid, the absence of procurement, and the absence of sweat equity. In other words, households did not need to be engaged in construction tasks, but did need to retain decision‐making authority with respect to both planning and financial management of construction activities. Again, there is a notable trend in the cases within this pathway: each was a repair and retrofit project that involved material distributions.

Surprisingly, household participation during the design phase of projects was not included in any of the three pathways to satisfaction. To reiterate, the study distinguished between involvement in and control over design decisions, the latter constituting in‐set membership. In line with past research (Kennedy et al., 2008), this study's findings indicate that household control of design decisions was not a necessary condition for satisfaction. More often, satisfaction was derived from the size and durability of shelter, irrespective of whether or not these decisions were made by the beneficiary. As one respondent put it: ‘We don't care that much on the physical aspects of the house, what we're after is a strong structure and its size; one that will fit our whole family’. This is not to suggest that beneficiary input was not important; rather, involvement in consultation meetings shaped desirable solutions across all of the projects.

The findings suggest that involvement in decisions, through either location selection or determination of aid, was present in all pathways. Pre‐determined aid criteria can undermine social connections, as targeting the most vulnerable families through a needs‐based framework has the potential to divide communities that are resentful about unequal aid distribution (Ong, 2015). Instead, local input can provide agency, cultivate social connections, and help to foster solidarity.

It is hypothesised here that government permitting may be an important condition for satisfaction, in part because of its ability to secure land tenure that establishes permanency and allows households to invest greater resources in shelter without fear of eviction. Government permitting played a role in shaping culturally appropriate and practical designs. For instance, one government official highlighted changes that he/she had recommended: ‘Some of the modifications that we were able to ask from the [NGO] were adding a kitchen sink to their design and providing a door on the side so that if the family would have more resources to add, for example a kitchen or a latrine, then it would be very accessible’. For this project, 87 per cent of households had expanded within a year of turnover—one of the highest rates across the projects reviewed—demonstrating that government participation had a tangible impact.

### Safe shelter design

**Figure 2:**
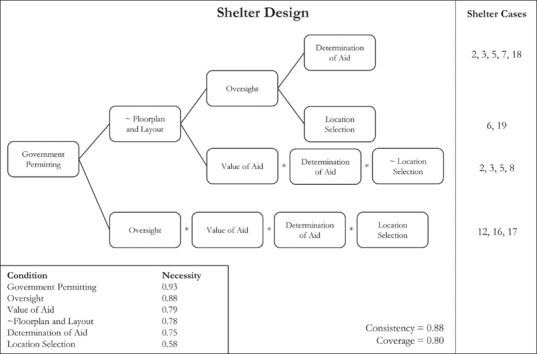
Shelter design pathways **Source:** authors.

Eleven of the shelter projects included more than five of the ‘8 Key Messages’, this study's measure of safe design. Observation of seven of the conditions was seen as constituting set membership, whereas incorporation of less than three messages denoted the absence of safe shelter design. Four pathways were identified with a collective consistency of ‘0.88’ and a coverage of ‘0.80’. Government permitting was found to be a necessary condition, with a necessity score of ‘0.93’. The first three pathways all included government permitting and the absence of household participation in floor and layout decisions. These pathways signify a high level of control over design by the implementing organisation and the local government. Figure 2 shows the pathways to safe shelter design.

The first pathway included five of the 11 cases that showed signs of safe design, whereas the second pathway included two cases. The first two pathways also included oversight during construction, with either determination of aid or location selection. Oversight was important in these pathways because of the ability to ensure that construction met intended guidance, regardless of whether design decisions were made by households or the implementing organisation. As all of the cases that fell into the first pathway were relocation projects, the damage levels experienced by the beneficiaries with regard to these projects were typically higher, leading to early assessments prioritising safety in shelter design. In contrast, the second pathway included the same conditions, except that location selection replaced determination of aid. The third pathway contained determination of aid, value of aid, and the absence of location selection. There was overlap in three of the cases for the first and third pathways. Value of aid emerged in place of oversight, suggesting that with sufficient resources, households were able to self‐select design components that were more robust. Labour participation was common across all cases in the third pathway, although project eight was distinct in that households were not involved in oversight processes during construction, explaining the reason for a separate pathway despite the other three cases appearing in the first pathway.

The last pathway encompassed three of the 11 cases with safe design elements, and included oversight, value of aid, determination of aid, and location selection. In comparison to the high level of organisationally imposed control during planning and design in the first three pathways, the last pathway demonstrates an alternative mechanism of achieving safe shelter design. While the first three pathways satisfy design via prescribed requirements, the last pathway does so largely through incentives. One of the cases in the last pathway relied on an owner‐driven model that utilised a three tranche conditional cash transfer. This delegated individual compliance with design standards to households, requiring that minimum standards were met before the next cash transfer was completed. A second project in this pathway placed responsibility for fulfilling design standards with local contracting teams. The last project in this pathway used volunteer labour to construct shelters. These shelter modalities, however, required significantly more financial resources per household: the average value of aid for these three cases was 33 per cent higher than the overall project average.

Government permitting surfaced as a necessary condition for safe design and oversight was nearly necessary, supporting past research that has identified the important role of oversight during construction (Jordan, Javernick‐Will, and Tierney, 2016). Government permitting is a logical participation condition to expect for design, yet little research has examined the role of local government in approving designs and synchronising settlement patterns. In one‐half of the pathways discovered, it was observed that a high value of assistance was required to achieve improved design. None of the repair and retrofit projects, all of low monetary value, achieved the design outcome, suggesting that a threshold of resources exists with respect to accomplishing a high level of design. This finding also indicates a need to examine more closely technical assistance programmes to understand resource constraints and other factors limiting the adoption and uptake of safer design principles.

## Discussion

The first phase of the study identified eight different project tasks that varied in their level of household participation. One‐half of the conditions pinpointed were in construction, suggesting that much of the participation in the observed shelter projects occurred in their later stages. The number of participation conditions should not be confused, though, with their relative importance, as demonstrated by the subsequent analysis (such as the significance of location selection).

Foremost, a high value of assistance emerged in a large number of the pathways to satisfaction and safe design, but its appearance was inconsistent. Alternative pathways where high monetary value does not manifest merit particular attention because they hold insights for humanitarian organisations faced with financial constraints. Household financial management was found to be vital for satisfaction in the absence of a high value of aid, showing promise for modalities that seek to support ‘self‐recovery’ and owner‐managed reconstruction. However, financial management did not appear in any of the pathways for safe design, a fact stemming perhaps from fewer resources being allocated to technical support for low value projects, such as material distributions.

Surprisingly, household participation in the design phase did not materialise in any of the pathways. While this finding contrasts with theoretically conceived outcomes of participation, it aligns with emerging empirical studies, which note that the importance of household control over designs often is overstated (Rand, Hirano, and Kelman, 2011). Participation during the planning phase happened almost universally across the two outcomes and hence one can conclude that earlier decisions were more influential in shaping shelter project outcomes. It may be logical to assume that design outcomes are tied to design decisions, but the analysis revealed that many of these decisions extend back to precursors concerning location selection and needs assessment. Location selection was frequently intractably linked to secure land tenure, a driver of household decisions to invest in shelter. Not surprisingly, the cases that exhibited these land tenure and location challenges were often situated in urban contexts. Connected to these contextual factors, government participation during the design phase was found to be important across all of the outcomes considered, suggesting a critical need to align shelter projects with broader recovery strategies emphasised by local governments.

In line with past research (see, for example, Vallance, 2015), labour participation was largely absent from the pathways. It was observed that significant sweat equity could lead to high satisfaction, but this participation was often highly intensive, amounting to hundreds of hours contributed across multiple months in the cases that led to high satisfaction. One beneficiary described how this labour investment resulted in satisfaction:

*We were more than happy to give a hand because those were our houses. We worked mornings and afternoons on the site. I was able to observe how the houses were built. I saw that the proportion of cement to gravel in each house was relatively higher. We really witnessed how the volunteers worked impressively on the houses. The materials were optimised and the gravel was all mixed compactly. The construction of the houses was not mediocre*.

This satisfaction was achieved at a cost, though, as many households that fulfilled these intensive sweat equity requirements had difficulty retaining paid employment to support household necessities during these labour periods. One beneficiary highlighted the impact of the requirements:

*My daughter was taken care of by my mother just so we can work there every day. We borrowed money and rice too, because we had no income during that time. It took us a month and two weeks to complete the 400 hours sweat equity by working eight hours daily, six days a week*.

Projects that mandated small labour hour requirements, typically between five and 40 hours, did achieve the same levels of satisfaction without as large of a burden on the beneficiaries. Furthermore, sweat equity or procurement did not appear in safe design pathways, reinforcing the conclusion that ‘sweat’ participation had little bearing on other project outcomes. While it is recognised that sweat equity can be a mechanism to promote ownership, too often the requirements hindered the economic recovery of households.

Of the conditions identified and analysed, location selection and determination of aid consistently appeared in both outcomes. The matter of where to build shelters, particularly in cases where physical hazards such as storm surge are present, is commonly overlooked. Not only does location spawn economic and social linkages, but in the case of the study outcomes, it was key to safe design, as households were more likely to invest in shelter if they knew that their presence was secure. Similarly, the participation of households in identifying needs was a precursor to establishing project modalities. For projects that did not conduct a formal needs assessment, the modalities were frequently poorly aligned with the shelter needs of households.

## Limitations

This study took significant steps to operationalise participation in post‐disaster shelter, but one should note several limitations of the work. Notably, participation alone does not explain all of the variation in the outcomes observed. Combining participation with other aspects of projects may yield additional insights. For instance, investigating participation and training together may be fruitful. Furthermore, a limitation of fsQCA is its inability to theorise vis‐à‐vis non‐observed cases in pathways, termed logical remainders. In addition, some conditions posited as influencing participation, satisfaction, and design did not vary across the case studies. These are domain conditions in qualitative comparative analysis. For example, cultural influences, such as the Filipino concept of ‘bayanihan’, or collective mutual effort, may have affected participation and the outcomes of interest; to know for sure requires cross‐cultural studies. The effect of these cultural influences is difficult to gauge, and, as some researchers have demonstrated in the wake of Haiyan (see, for example, Eadie and Su, 2018), stereotyped cultural values may have an overstated potential impact on recovery processes, although additional research is needed to discover if this is so. Nonetheless, this paper has taken a substantial stride in advancing systematic cross‐case analysis of post‐disaster shelter.

## Conclusion

Participation frameworks are plentiful in the literature, but research is sparse on operationalising and measuring participation in post‐disaster shelter projects. To address this lacuna, this study examined 19 shelter projects following Typhoon Haiyan in the Philippines, adopting a grounded approach to pinpoint forms of participation that surface in shelter projects. The analysis identified eight types of participation in project tasks: (i) determination of aid; (ii) location selection; (iii) floorplan and layout; (iv) government permitting; (v) sweat equity; (vi) material procurement; (vii) financial management; and (viii) oversight. These tasks were found to be aligned with the planning, design, and construction phases of shelter projects. The resulting typology of participation conditions affords a way to assess and operationalise participation in post‐disaster shelter projects, answering calls to specify and define what participation means in a project environment (Davidson et al., 2007).

Using the participation conditions ascertained in the first phase, the study then explored their relative importance in the generation of two shelter project outcomes: household satisfaction with shelter; and safe shelter design. Early participation in planning was found to be essential, but projects could lead to satisfaction through either the high value of aid supplied, or alternatively, through household management of project finances. Household participation during the design phase did not appear in satisfaction pathways, aligning with past work that suggests that involvement is necessary, but that control is not required to achieve satisfaction outcomes (Kennedy et al., 2008; Rand, Hirano, and Kelman, 2011). Safe shelter design was found to be accomplished primarily through organisational and governmental control over project processes, although the study did reveal a limited number of cases that resulted in a high level of design owing to household participation during planning and construction. This builds on previous theory that has posited that control over technical decisions by non‐technical individuals may lead to poor design outcomes (Khwaja, 2004).

Several lessons can be drawn from this research. First, discourse on participation in humanitarian shelter projects should recognise the diversity of tasks that constitute participation. ‘Sweat’ participation often is discounted as insignificant, but, as demonstrated, there is potential for it to further project goals, if paired appropriately with other types of participation. Organisations that seek to employ such strategies should recognise that this type of participation has the potential to become tyrannical in nature if adequate evaluations of time and resources contributed by beneficiaries are not undertaken (Cooke and Kothari, 2001). More broadly, it is important for organisations to appreciate the limits of participation in humanitarian programming. Assuming that short‐term project participation will lead to community ‘empowerment’ is a gross oversimplification of complex community social dynamics. While participation in shelter projects can be an impetus for social change, its impact will be short‐lived unless it is linked to long‐term capacity‐building efforts.

Second, as illustrated by the equifinality of the solutions to attaining outcomes, there is no one answer concerning participation. Many of the combinations found included differing types of participation, yet reached the same outcome. Organisations should tailor household participation to their individual modality of delivering shelter assistance. Successful participation strategies will be linked to site‐specific conditions, such as land tenure, level of damage, broader settlements planning, and pre‐existing capacities. For humanitarian policy, this means avoiding the urge to provide blanket participation strategies for programmes.

Finally, this research challenges previous conceptualisations of participation (Arnstein, 1969; Choguill, 1996), principally that informing and consulting processes do not yield value. Rather than idealising participation as beneficiary control, it should be viewed as the collaborative pursuit of project aims and tasks. Successful participation should not be judged by how much control is relinquished to beneficiaries, but rather by the extent to which project outputs meet community priorities and needs.

## Annexes

### Annexe 1. Condition calibrations

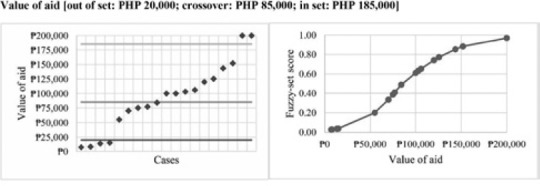

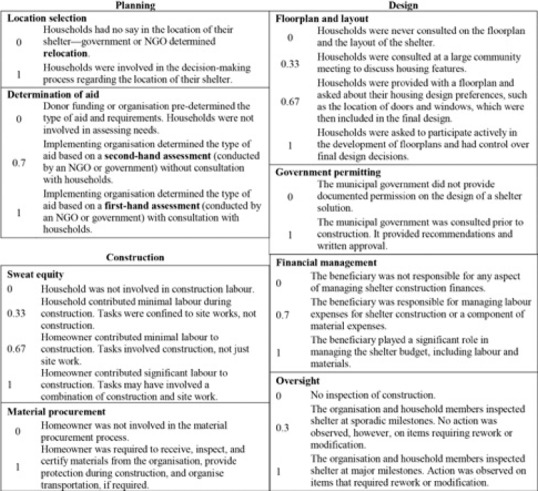

### Annexe 2. Outcome calibrations

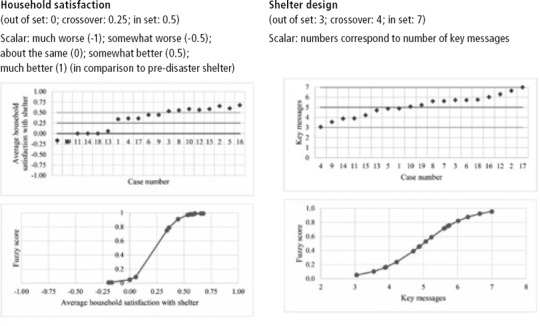

### Annexe 3. Truth table

**Table:** 

Community	Value of aid	Location selection	Determination of aid	Floorplan and layout	Government permitting	Sweat equity	Procurement	Financial management	Oversight	Satisfaction	Design
Okoy	0.65	1	0.7	0.33	1	0.33	0	0	0.3	0.75	0.46
Maricaban	0.97	0	0.7	0	1	1	0	0	1	0.99	0.92
Poblacion	0.88	0	1	0	1	1	0	0	1	0.97	0.75
Sungko	0.04	1	1	1	0	0.33	0	0.7	0	0.79	0.05
Sillon	0.63	0	1	0	1	0	0	0	1	0.99	0.45
Kangkaibe	0.49	1	0	0	1	0	1	0	1	0.91	0.75
Tagpuro	0.39	0	0.7	0	1	0	0	0	1	0.01	0.72
Pago	0.97	0	0.7	0	1	1	0	0	0.3	0.98	0.71
New Kawayan (101)	0.03	1	1	1	0	0	0	0.7	1	0.91	0.10
Bagacay (93)	0.74	1	0	0	1	0.33	0	0	0.3	0.98	0.53
San Agustin	0.61	1	0	0	0	0.67	0	0	0	0.05	0.16
San Jose (83C)	0.61	1	1	0.67	1	0	0	0	1	0.98	0.88
Magallanes (52)	0.20	1	1	0.67	1	0.33	1	1	1	0.09	0.39
San Jose (85)	0.03	1	1	1	0	0.33	1	1	0.3	0.05	0.16
Hiabangan	0.04	1	1	1	0	0.33	0	1	0	0.98	0.24
Sagkahan (62)	0.77	1	1	0.67	1	0	1	0	1	0.99	0.82
Sulangan	0.85	1	0.7	0.67	1	0.33	1	1	1	0.79	0.95
Cogon	0.41	0	0.7	0	1	0	0	0	1	0.05	0.76
Cantahay	0.33	1	0	0.33	1	0.33	1	0.7	1	0.01	0.59

### Annexe 4. Simplifying assumptions

**Table:** 

	Satisfaction	Design
Value of aid	Present	Present
Location selection	Present	Present
Determination of aid	Present	Present
Floorplan and layout	Present	Present or absent
Government permitting	Present	Present
Sweat equity	Present	Absent
Procurement	Present	Present
Financial management	Present	Present or absent
Oversight	Present	Present

**Source (annexe section)**: authors.

## Acknowledgements

This material is based on work supported by the National Science Foundation (grant number: 1434791) and the United States Agency for International Development's Office of US Foreign Disaster Assistance (award number: AID‐OFDA‐G‐16‐00048), as well as by the Nicolas R. and Nancy D. Petry Fellowship in Construction Engineering and Management. Any opinions, findings, conclusions, or recommendations expressed in this material are those of the authors and do not necessarily reflect the views of the funding agencies.

Lastly, thanks are extended to Hannah Moench for her contribution to the qualitative comparative analysis, to Marielle Bacason, Jairus Josol, Lebeth Manguilimotan, and Phoebe Tabo for their assistance in collecting the data, and to Shaye Palagi for her help in assembling the case studies.

## Endnotes

1 One should note that the term ‘shelter’ is used here to describe built household spaces; it is recognised, though, that the literature interchangeably uses ‘shelter’, ‘housing’, and ‘habitat’.
